# Effects of a Muscle Relaxation Technique on Catatonia Symptoms Associated With Schizophrenia: A Case Report

**DOI:** 10.7759/cureus.66972

**Published:** 2024-08-15

**Authors:** Tomoki Kakehashi, Masaaki Nakajima

**Affiliations:** 1 Physical Therapy, Minamiawaji Hospital, Minamiawaji, JPN; 2 Physical Therapy, School of Health Science and Social Welfare, Kibi International University, Takahashi, JPN

**Keywords:** beta-endorphin, activities of daily living, peroxisome proliferator-activated receptor gamma coactivator 1-alpha, squeeze hold, catatonia

## Abstract

Catatonia is characterized by the loss of voluntary control over the workings of the mind and body. It disrupts daily life by manifesting as idle posture, heightened muscle tone, and repetitive purposeless movements. However, specific physiotherapy methods addressing these symptoms are yet to be established. This case report describes a 63-year-old man hospitalized for schizophrenia who was then diagnosed with stuporous catatonia based on the Diagnostic and Statistical Manual of Mental Disorders, Fourth Edition, Text Revision (DSM-IV-TR) criteria, characterized by catalepsy, mutism, and difficulty performing daily activities. This case report aimed to evaluate the effectiveness of a specific muscle relaxation technique, squeeze-hold (SH), in treating catatonia associated with schizophrenia and its impact on daily activities. The patient exhibited catalepsy, mutism, and difficulty in performing daily activities. The SH technique employed temporarily obstructs muscle blood flow to induce ischemia, resulting in the relaxation of vascular smooth muscle due to CO_2_ retention. Furthermore, shear stress upon reperfusion stimulates nitric oxide production in the vascular endothelium, enhancing blood flow. Following weekly SH on the bilateral thighs, the muscle tone in the lower extremities was alleviated within two weeks, and the patient no longer required a wheelchair by the eighth week. In addition, responsiveness to verbal commands improved. As muscle tone in the lower limbs improved, the patient regained ambulation, and his improved responsiveness facilitated independent eating during activities of daily living (ADLs), potentially enhancing motivation and spontaneity. These findings suggest that muscle tone relaxation due to enhanced blood flow and increased CO_2_ concentration from blood flow restriction may have promoted β-endorphin secretion, thereby improving symptoms via brain-derived neurotrophic factor expression through PGC-1α activation. In conclusion, the SH muscle relaxation technique effectively alleviated catatonic symptoms, and improved muscle tone and daily functioning in patients with schizophrenia-associated catatonia. These findings suggest that this physiotherapy approach may be a valuable addition to catatonia treatment, potentially contributing to physical and psychiatric rehabilitation. This case report illustrates the efficacy of a muscle-tone-focused treatment approach in physical therapy for catatonia and posits its contribution to the reacquisition of psychiatric function and ADLs.

## Introduction

Catatonia is a syndrome associated with various diseases and has an estimated prevalence of approximately 35% among patients with schizophrenia [1]. This syndrome is characterized by immobility, repetitive movements, difficulty in voluntarily controlling body movements, and significant muscle tension. The prognosis is often poor as these symptoms persist, leading to deteriorating nutritional status and reduced activities of daily living (ADL). Although pharmacological interventions and electroconvulsive therapy (ECT) treat catatonia effectively, the recurrence rate is high, and the symptoms can become chronic [2]. Furthermore, even if symptoms improve and spontaneity is regained, the transition from catatonic stupor to aggressive behavior remains a potential risk. This highlights the need for continuous monitoring and comprehensive treatment approaches. In the rehabilitation field, oxidative stress generated by exercise promotes the secretion of peroxisome proliferator-activated receptor gamma coactivator 1-alpha (PGC-1α) and β-endorphin and induces the expression of brain-derived neurotrophic factor (BDNF) [3,4]. These factors may have a beneficial effect on behavioral disorders and emotional regulation in patients with catatonia [3,4]. However, patients with catatonia often struggle to engage in voluntary exercise, which complicates the implementation of exercise therapy. One potential solution is squeeze-hold (SH) therapy, a technique that involves compressing the muscles to temporarily restrict blood flow and then releasing pressure to enhance blood flow [5]. This method elevates local CO_2_ concentration and reduces pH, potentially mimicking the effects of exercise and may be effective in treating catatonia [5]. This case study aimed to provide preliminary evidence for the potential use of SH in treating catatonia, particularly in patients who struggle with voluntary exercise. By documenting the outcomes of this novel approach, we aimed to lay the groundwork for future comprehensive studies on the efficacy of SH in catatonia management. This report documents the application of SH in a patient with catatonia, who demonstrated significant improvement.

## Case presentation

A 63-year-old man was hospitalized for schizophrenia. The patient had been hospitalized in 2003 due to increased anxiety symptoms, which led to difficulty in eating. In 2018, the patient began exhibiting violent behavior due to irritability, resulting in multiple hospitalizations and outpatient visits to our hospital. The patient presented with suicidal ideation, fatigue, decreased personality function, and poor impulse control. The variability and duration of these symptoms aligned with the Diagnostic and Statistical Manual of Mental Disorders, Fifth Edition (DSM-5) diagnostic criteria for schizophrenia. Based on these symptoms and a specialist's assessment, the patient was diagnosed with schizophrenia on May 26, 2018.

At the time of hospitalization in 2018, the patients tended to seclude themselves in their rooms but were independent of ADLs. In June 2021, approximately three years after hospitalization, the patient began to show a decline in ADLs (specifically in excretion). In April 2022, approximately three years and eight months after hospitalization, the patient developed severe muscle tension, along with a significant decrease in voice volume and occasional delusional speech. By January 2023, nearly 4.5 years after hospitalization, speech was almost completely absent, with a marked decrease in vitality, further decline in ADLs, and difficulty in eating. In February 2023, walking gradually became difficult and the patient began using a wheelchair. On September 26, 2023, approximately five years after hospitalization, the patient was diagnosed with catatonia based on the DSM, Fourth Edition, Text Revision (DSM-IV-TR) diagnostic criteria. This diagnosis was made based on stupor, immobility, negativism, mutism, posturing, and bizarre repetitive movements. We used the Catatonia Rating Scale (CRS) to evaluate catatonic symptoms [6]. The assessment was conducted by a physical therapist with three years of experience in psychiatric rehabilitation. To ensure the quality and consistency of the evaluation, we adhered to the following procedures. Evaluator training - for specific symptom evaluation, we used symptom explanation videos of the Bush-Francis CRS (BFCRS) as the reference material. This enabled the accurate identification and assessment of each symptom. Use of reference materials and expert supervision - all evaluations were conducted under the guidance and supervision of a psychiatrist. Notably, all the symptoms mentioned, except for the initial diagnosis of schizophrenia, appeared after hospitalization in 2018. The patient was hospitalized when their condition deteriorated to the point where eating and taking medication became difficult. Before the diagnosis of catatonic stupor, the patient experienced progressive difficulty in walking and a decline in ADL. Initially, Parkinsonism was suspected, and levodopa was prescribed, but proved ineffective. Subsequently, treatment with lorazepam (0.5 mg; six tablets), a benzodiazepine, was initiated. Approximately six months later, there was no improvement in his ADLs, and the patient remained immobile. The patients’ daily activities were significantly affected by various catatonic symptoms. Catatonic immobility (akinesia), characterized by a lack of movement, results in an overall inability to perform actions, whereas stereotypy causes the patient to persist with repetitive, meaningless movements. Both these symptoms significantly impaired the patient's ability to perform ADLs, and mutism made communication difficult, severely limiting their ability to interact with others. The patient's tendency to refuse assistance is also hindered by efforts to help him regain his daily living skills. He also showed strong resistance to accepting the necessary assistance in nursing and caregiving situations and was forced to live in a wheelchair. Aerobic exercise (using an ergometer) was prescribed for rehabilitation but was difficult to implement because of the patient’s stupor.

Intervention

**Figure 1:**
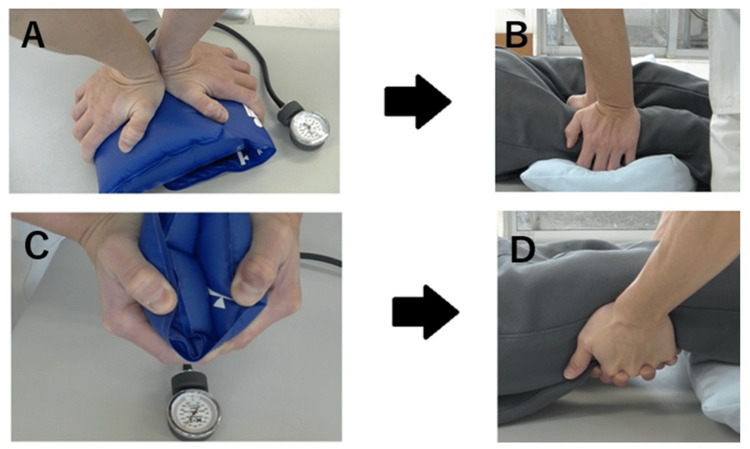
Squeeze-hold technique (A, C) The cuff was inflated to 100 mmHg, and the degree of the pressure load was confirmed via manual squeezing. (B) Pressure was applied vertically above the quadriceps and held for 20 s. This procedure was performed on the entire quadriceps. (D) The hamstrings were squeezed by pinching them between the palms of both hands and holding them for 20 s. This procedure was performed on all hamstring muscles. Image Courtesy: Author

The patient was treated with manual SH techniques on the quadriceps and hamstrings once a week while lying in a supine position. The SH technique involves squeezing the target muscle with both palms and holding for 20 s [5]. The compression intensity was set at approximately 200 mmHg using a cuff (Sankei, 19.5 cm wide) inflated to 100 mmHg (Figure 1) [5]. Although progressive relaxation is not the primary approach to treating catatonia, its regular practice can be beneficial as part of a broader multidisciplinary approach. Importantly, clinical evidence for the effectiveness of progressive relaxation, proposed by Jacobson or Schultz, for catatonia is limited. The SH method, performed by physical therapists, is an effective treatment; however, it only plays a supplementary role. Therefore, a more comprehensive and multidisciplinary approach is necessary [7]. Electroconvulsive therapy (ECT) was also considered a treatment option, but it remained at the discussion stage because it was not available at our facility. Catatonic syndrome does not appear only in patients with schizophrenia, and it is necessary to consider the possibility of an underlying organic pathology. Therefore, we have re-recognized the importance of a holistic approach and multidisciplinary teamwork and felt the need to apply this to future cases.

Evaluation

Quadricep muscle tone was assessed in the lateral position using the Modified Ashworth Scale (MAS) (Table 1) [8]. Responsiveness was measured by recording the reaction time to verbal instructions from the therapist, based on the methodology of mental chronometry [9]. Specifically, the patient was instructed to “please stand up” five times, with a 30-second interval between instructions, and the reaction of the patient was observed (Table 2). ADLs were evaluated using the functional independence measure (FIM) in ward life [10]. During the intervention stage, when the patient lacked voluntary movement, the technique was adapted passively. This involved the therapist applying pressure to the target muscle groups using both palms, maintaining the pressure for 20 s, and then releasing them. The compression intensity was set at approximately 200 mmHg and calibrated using a cuff inflated to 100 mmHg. This adapted method differs substantially from Jacobson's original technique, which requires active patient participation [11]. Importantly, we considered introducing the standard Jacobson Progressive Relaxation technique as a potential follow-up intervention once the patient regained spontaneity and voluntary movement.

**Table 1:** Modified Ashworth Scale for grading spasticity

Grade	Description
0	No increase in muscle tone
1	Slight increase in muscle tone, manifested by a catch and release or by minimal resistance at the end of the range of motion when the affected part(s) is moved in flexion or extension
1 +	Slight increase in muscle tone, manifested by a catch, followed by minimal resistance throughout the remainder (less than half) of the ROM
2	More marked increase in muscle tone through most of the ROM, but affected part(s) moved easily
3	Considerable increase in muscle tone, passive movement difficult
4	Affected part(s) rigid in flexion or extension
9	Unable to test

**Table 2:** Assessing the response to verbal instructions for standing up A step was assigned based on the state of response to verbal instructions. In Step 1, the movement leading to standing up (pulling the feet back and leaning the trunk forward) was observed and judged. In Step 3, the patient was able to stand up completely.

Step	Response
0	No response
1	Initiates movement in a seated position (e.g., shifts weight, leans forward) but does not rise
2	Raises hips from the sitting surface but cannot complete standing
3	Stands up completely

At the three-month follow-up after the eight-week intervention, we observed that the patient developed impulsivity and stern facial expressions, while rigidity persisted. However, there was only a slight improvement in stereotypy and mutism. The CRS scores reflected these changes; the score was 60 points at the start of the intervention, it decreased to 52 points at the final evaluation (eight weeks), and it further reduced to 47 points at the three-month follow-up. This gradual decrease in CRS scores indicated a modest improvement in overall catatonic symptoms over time, including mutism, although some symptoms persisted, and new behaviors emerged (Table 3) [6].

**Table 3:** Catatonia Rating Scale (CRS) The CRS scores from the pre-intervention assessment to the midpoint and end of the intervention were recorded. Follow-up scores were recorded three months after the end of the intervention.

CRS	Before intervention	Mid-intervention	After intervention	Follow-up
Groping	5	5	5	1
Stereotypies	5	5	5	1
Iterations	1	1	1	1
Verbigerations	1	1	1	1
Grimacing	1	1	1	5
Jerky movements	1	1	1	2
Posturing	5	5	5	1
Rigidity	5	5	5	5
Blinking	1	1	1	1
Motor excitement	1	1	1	1
Motor inhibition	4	3	3	3
Exaggerated responsiveness, copying	1	1	1	1
Gegenhalten	5	5	3	4
Parakinesias	5	4	4	3
Waxy flexibility	5	5	5	2
Mutism	5	4	4	4
Mannerisms	1	1	1	1
Automatic	1	1	1	1
Negativism	5	4	2	5
Impulsivity	1	1	1	3
Rituals	1	1	1	1
Total	60	56	52	47

**Figure 2:**
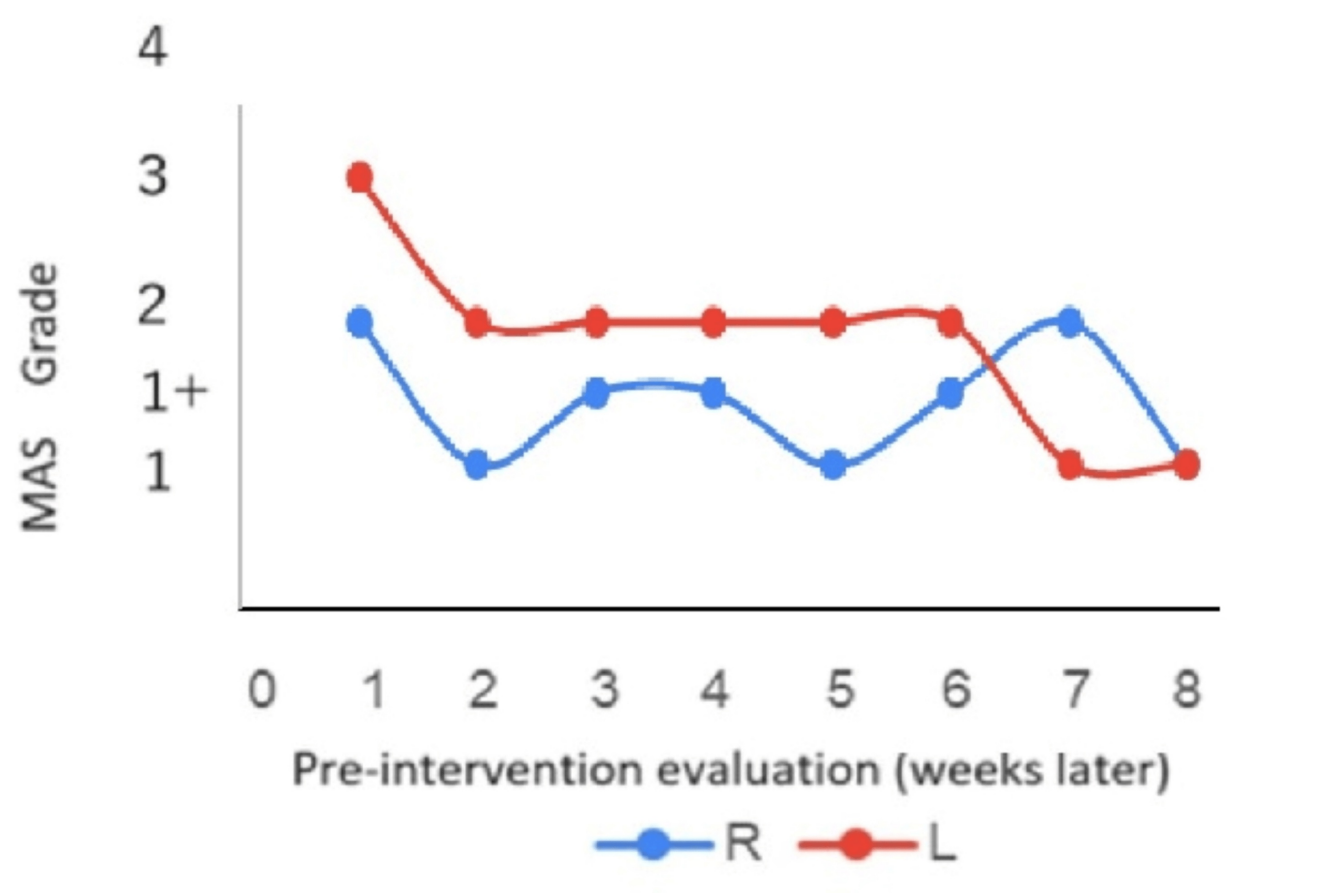
Changes in quadriceps muscle tone (MAS) before SH intervention The change in the MAS score before each week of intervention is shown, with the start of the intervention marked as week 1. Although differences and some fluctuations were observed between the left and right sides, the final pre-intervention evaluation indicated a decrease in the MAS score by at least one level. SH - squeeze hold

**Figure 3:**
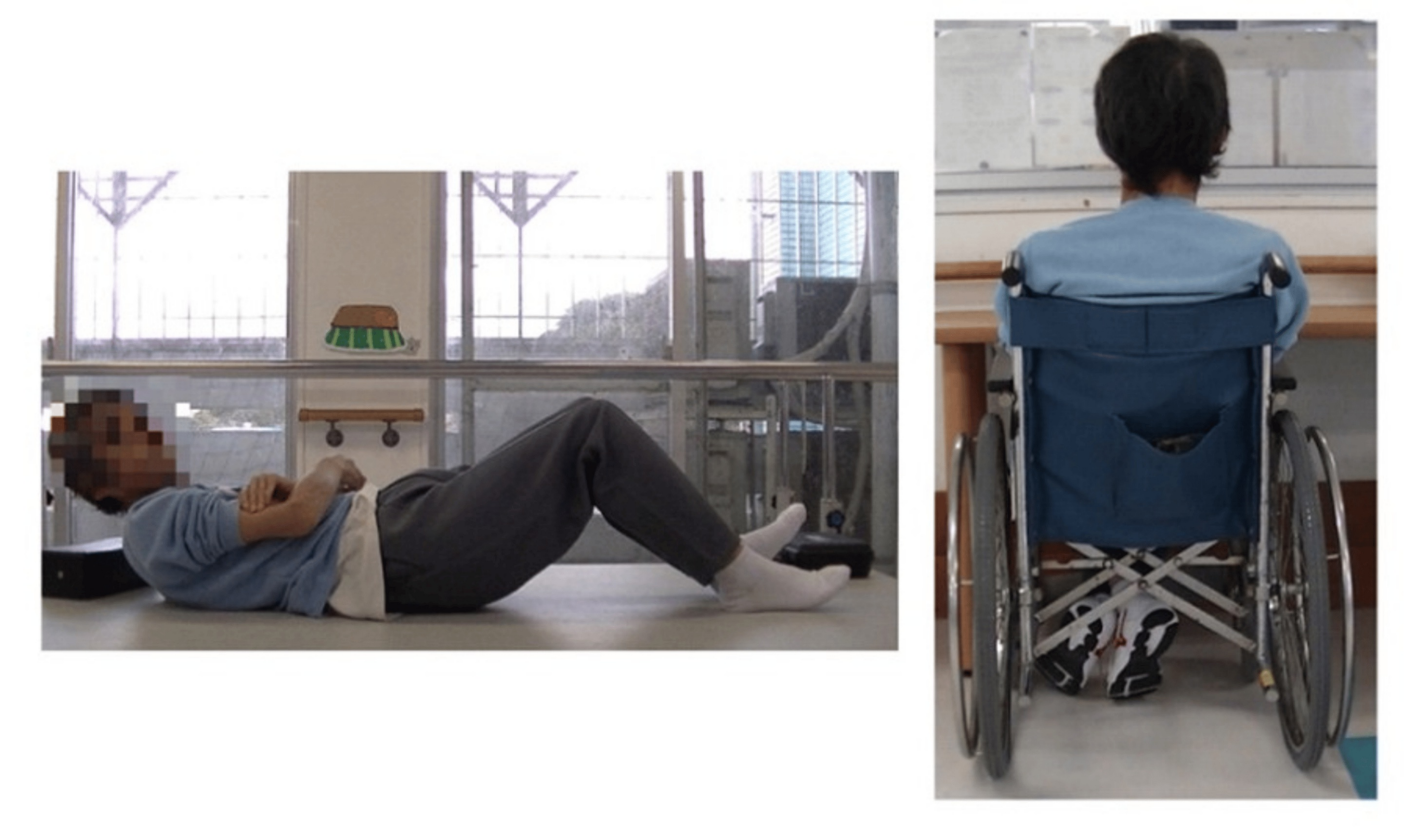
Supine position and wheelchair seated position before the initiation of intervention The patient exhibited high muscle tone throughout the body and was prone to flexion even in the supine position. The hands of the patient constantly scratched the upper arms adjacent to the body (stereotypy). This behavior persisted even when the patient was seated in a wheelchair. Image Courtesy: Author

**Figure 4:**
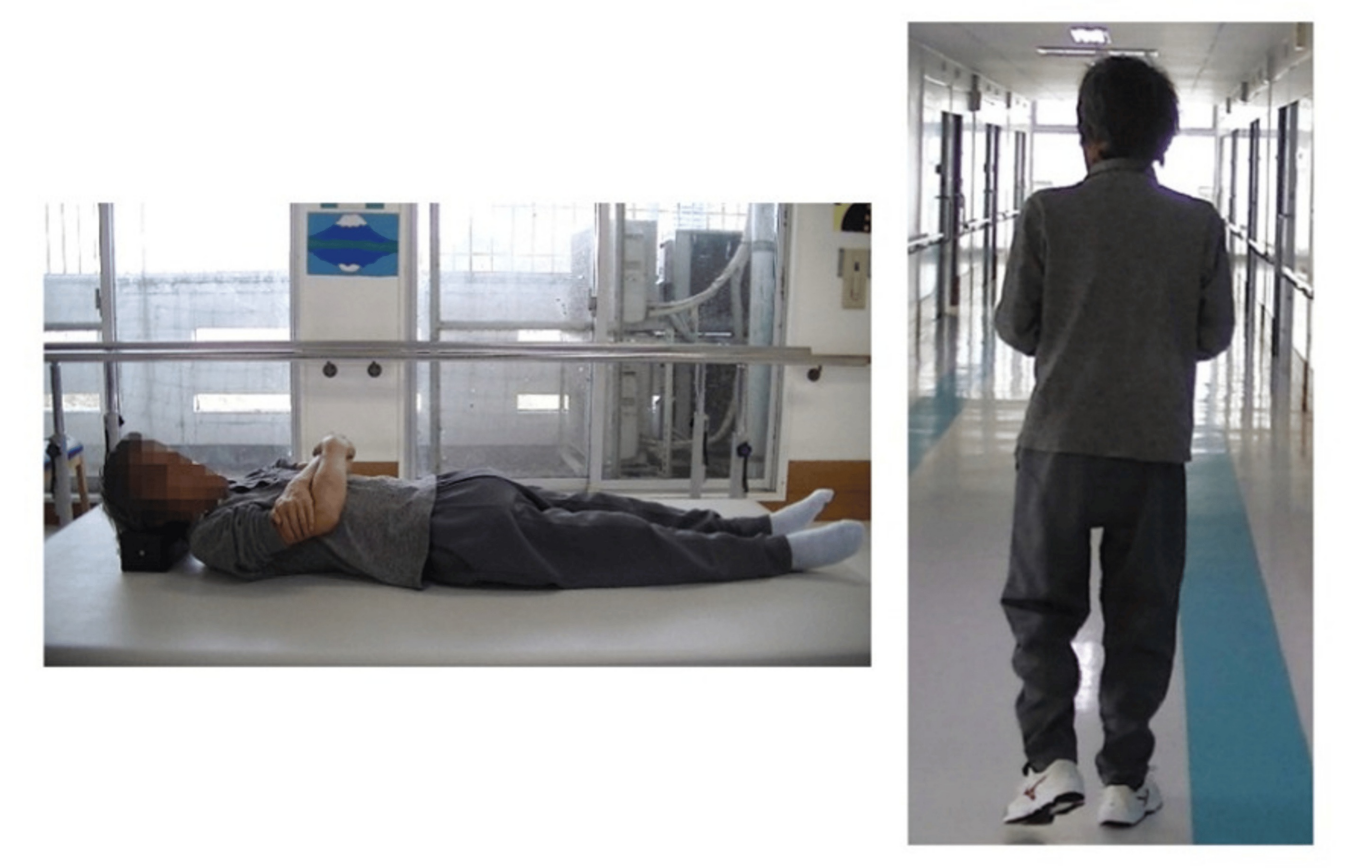
Supine posture and walking behavior upon walking acquisition Muscle tension in the supine position was alleviated, allowing the patient to relax the body easily. Stereotypy was reduced during walking, and the patient could walk independently with guidance or partial assistance. Image Courtesy: Author

In the MAS evaluation, quadriceps muscle tone decreased by at least one level out of five by the second week after the intervention began (Figure 2). In the assessment of responsiveness, the patient could stand up from the second week after the intervention began, although reaction times varied (Table 4). After six weeks of intervention, the FIM score for eating improved from 1 to 3 points, and by the eighth week, the patient had progressed from using a wheelchair to walking with assistance, with the FIM score improving from 1 to 4 points (Figures 3, 4).

**Table 4:** Responses to verbal commands and changes in activities of daily living (ADLs) *Gradation based on “Judgment of Response to Prompts to Stand Up” in Table 2. Blank indicates no response or changes. Daily living activities, particularly eating and moving, which are problematic in nursing and care situations, were evaluated.

Progress	Timing	Steps*	Number of instructions	Time required (seconds)	ADL
Week 1	pre	0			No change (full assistance)
	post	1	3	10.37	
Week 2	pre	0			
	post	3	1	4.71	
Week 3	pre	0			
	post	3	3	5.38	
Week 4	pre	0			
	post	3	1	16.91	
Week 5	pre	0			Walks independently infrequently (No change in FIM)
	post	3	2	3.71	
Week 6	pre	0			Eats food independently (FIM diet: 1 → 3)
	post	3	5	2.67	
Week 7	pre	0			
	post	0			
Week 8	pre	0			Can walk with minimal assistance (FIM diet: 3 → 5) (FIM mobility: 1 → 4)
	post	1	5	13.39	

A follow-up evaluation was conducted three months after the intervention. Improvements in eating and mobility persisted, maintaining the same level as that immediately after the intervention. However, other ADLs require assistance. The MAS scores were as follows: quadriceps, 0 on the right and 1 on the left. The patient's response to verbal instructions improved, completing the sit-to-stand task with a single verbal cue (Stage 3) with a reaction time of 4.63 seconds.

## Discussion

Summary of results

Exercise therapy was conducted one to two times per week. Aerobic exercises included assisted walking and using a cycle ergometer, although the patient required extensive assistance for both activities. Stretching exercises were also performed. Notably, the level of assistance required for these exercises was significantly high. Muscle tone improved within two weeks, and eating and walking abilities improved within six weeks. By the eighth week, the patient could walk without the need for a wheelchair. The MAS assessment showed an improvement from 3 to 1 in the left lower limb and 2 to 1 in the right lower limb. As the muscle tone in the lower limbs improved, the patient could walk with partial assistance. Enhanced responsiveness contributed to an improved ability to feed oneself and positively influenced spontaneity and motivation. Throughout the intervention period, the patient was consistently prescribed six tablets of lorazepam (0.5 mg), a benzodiazepine. One month after the intervention, amantadine hydrochloride (50 mg, four tablets) was added to the regimen and then maintained for the remainder of the intervention period. There was no change in the medication status for psychiatric symptoms during the intervention period, indicating that SH was effective in reducing muscle tone and improving spontaneity in catatonia.

Notably, the SH technique used in this study differs significantly from Jacobson's Progressive Muscle Relaxation. While both aim to achieve muscle relaxation, our method does not require active patient participation and instead relies on the physiological effects of ischemia-reperfusion. The SH technique was initially developed and reported by Nakajima et al. [5], who observed muscle relaxation effects in a case study. Although this method has been previously documented, its validation is currently limited to small-scale studies. The primary investigator in this study received direct training in using this technique from Nakajima and applied it accordingly.

Exercise therapy for catatonia

During rehabilitation, aerobic exercise is used to improve psychiatric symptoms and maintain activity levels [12]. The decrease in intramuscular pH and the increase in CO_2 _concentration induced by exercise promote the secretion of β-endorphin in the hypothalamus [4,13]. Endorphins are involved in the regulation of dopamine in the hypothalamus and striatum, potentially addressing the dopamine dysfunction underlying catatonia [14]. Proper regulation of β-endorphin and dopamine levels may improve motor disorders, motivation, and emotional disturbances in patients with catatonia. However, because patients with catatonia often struggle with voluntary muscle activity, the implementation of aerobic exercise is challenging, necessitating alternative methods.

Endorphins

β-endorphin is a peptide secreted in the brain, primarily from the anterior pituitary gland and hypothalamus, in response to pain and stress [13,15]. It activates the reward pathways and regulates dopamine levels, thereby contributing to stress relief and anxiety suppression [14,15]. Additionally, β-endorphin impacts the immune system and regulates cytokine production [13,15].

Considerations for the effectiveness of SH

**Figure 5:**
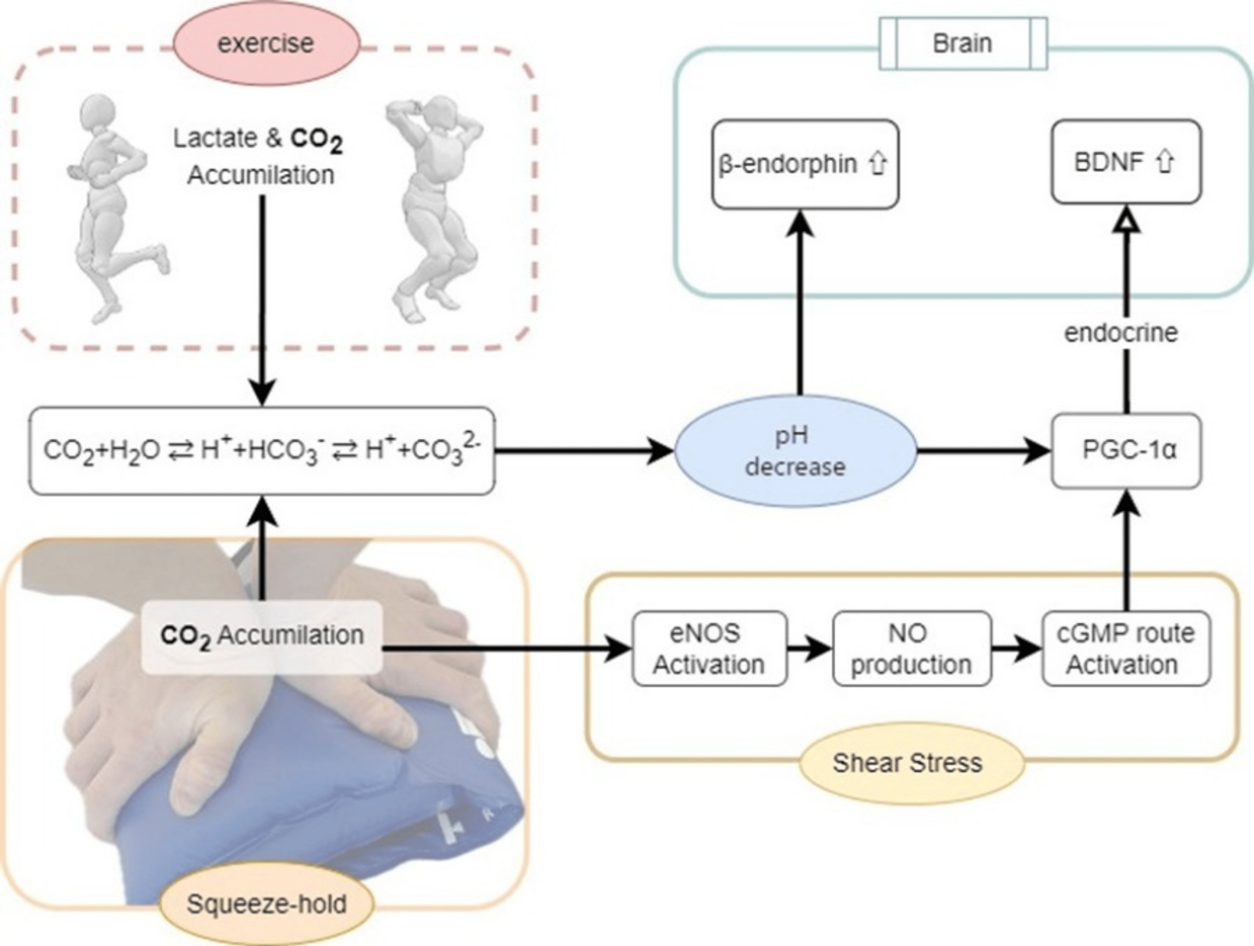
Mechanism of endorphin secretion, and BDNF expression by SH. Exercise induces a decrease in pH owing to the accumulation of lactic acid and an increase in CO_2_ concentration. Similarly, the squeeze-hold (SH) intervention increases CO_2_ concentration in muscles, resulting in physiological changes akin to those caused by exercise. Decreased pH promotes the secretion of β-endorphins in the brain. Exercise and SH induce shear stress in vascular endothelial cells, which promotes PGC-1α expression through the activation of the cGMP cycle via NO. Additionally, PGC-1α promotes BDNF production. These changes in the brain neuroenvironment induced by SH may improve the symptoms of catatonia. Abbreviations: CO₂ (carbon dioxide), H₂O (water), H⁺ (hydrogen ion), NO (nitric oxide), HCO₃⁻ (bicarbonate ion), CO₃²⁻ (carbonate ion), eNOS (endothelial nitric oxide synthase), cGMP (cyclic guanosine monophosphate), β-endorphin (beta-endorphin), pH (potential of hydrogen), PGC-1α (peroxisome proliferator-activated receptor gamma coactivator 1-alpha), BDNF (brain-derived neurotrophic factor)

SH increases intramuscular CO_2_ concentration and decreases pH [5]. This change relaxes the vascular smooth muscle layer of the vessels in the muscles and promotes blood flow [16]. Additionally, nitric oxide (NO), a vasodilator, is released temporarily after the termination of SH, further increasing blood flow [17]. Nakajima et al. [5] demonstrated that SH improves muscle tone in the short term. This improvement in muscle tone likely contributed to the observed improvement in catatonic symptoms. A decrease in pH promotes the secretion of β-endorphins in the brain, which can improve mood and motivation [4]. Taylor et al. demonstrated a strong correlation between acidosis markers and β-endorphin levels. They found significant correlations between β-endorphin concentrations and acidosis indicators, such as pH (r = -0.94), PCO_2_ (r = -0.85), HCO_^3^_^-^ (r = -0.88), base excess (r = -0.94), and lactate (r = 0.89) [4]. These correlations suggest that changes in acidosis may be involved in β-endorphin secretion, although the exact quantitative relationship and detailed mechanisms in the context of catatonia remain unclear. The NO released upon blood flow restoration after SH activates the cyclic guanosine monophosphate (cGMP) signaling pathway [18]. This cGMP pathway activation may contribute to the activation of PGC-1α [19]. Furthermore, PGC-1α plays a crucial role in hippocampal synapse formation [20]. Theoretically, this activation of PGC-1α may promote BDNF production in the brain and influence dopamine regulation through BDNF [3,17]. However, the direct role of this mechanism in catatonia or SH therapy has not been fully verified and remains a subject for future research. Additionally, the exhilarating sensation experienced upon termination of SH is hypothesized to result from the activation of the reward system that promotes β-endorphin secretion [5]. Therefore, it is possible that the motor symptoms and loss of motivation associated with catatonia may improve through the accumulation of CO_2_ by SH and the promotion of NO and β-endorphin expression (Figure 5). However, these mechanisms are largely inferred from studies in different contexts, and direct evidence in catatonia is limited. Alternative explanations, such as spontaneous remission or delayed medication effects, could not be ruled out in this single-case study.

Study limitations

This study had several limitations. The generalizability of our results is severely limited as a case report describing a single patient. We recognize this as a major limitation of the current study. Although a large-scale study involving numerous patients with catatonia would have been ideal, we understand the practical challenges of recruiting such a specific patient population. Instead, we conducted a small series of case studies to build a more diverse patient base. A crucial limitation is the lack of quantitative measurements of physiological indicators. This absence prevents the establishment of a direct causal relationship between the physiological changes caused by CO_2_ accumulation and symptom improvement. In future studies, we will strive to incorporate more accessible quantitative measures, such as heart rate variability, blood pressure changes, and standardized CRSs. While comprehensive blood tests for β-endorphin and PGC-1α levels may not be feasible in our current setting, we will explore collaboration with local laboratories to conduct basic blood work where possible. The degree of pressure loading during SH was premeasured using a cuff; however, more accurate measurements are important. In future studies, we plan to use more precise pressure-monitoring devices to ensure optimal and consistent pressure application. Additionally, to address the lack of long-term data, we plan to design future case studies with extended follow-up periods within the constraints of our clinical setting. Although animal studies and neuroimaging are beyond our current scope, we will thoroughly review and incorporate the findings from studies conducted by other research groups to improve our understanding of the underlying mechanisms. We also seek to establish collaborations with other institutions that have these capabilities if opportunities arise. By addressing these limitations to the best of our abilities in future research, we aim to provide more robust findings on the efficacy of SH in treating catatonia while acknowledging the inherent constraints of our research environment.

## Conclusions

This case report suggests that the SH technique may have potential benefits as part of a comprehensive, multidisciplinary approach to treating catatonia associated with schizophrenia. SH showed effects similar to those of exercise therapy and can be particularly beneficial for patients with difficulty performing spontaneous movements. However, it is crucial to recognize that catatonia is a complex syndrome requiring individualized treatment, and SH should be considered a potential complementary technique rather than a standalone treatment.

Although these findings are promising, they serve as a basis for further investigation, rather than being definitive conclusions. Future research should explore how the SH technique can be integrated into comprehensive treatment plans, considering the full spectrum of available interventions and the need for personalized care. Further studies are warranted to verify the effect of SH in a larger patient population with catatonia, elucidate its mechanism, and investigate its potential benefits when combined with other treatments, such as pharmacotherapy and ECT.
